# Ca^2+^/Calmodulin-Dependent Protein Kinase Kinase Is Not Involved in Hypothalamic AMP-Activated Protein Kinase Activation by Neuroglucopenia

**DOI:** 10.1371/journal.pone.0036335

**Published:** 2012-05-10

**Authors:** Junji Kawashima, Thierry Alquier, Youki Tsuji, Odile D. Peroni, Barbara B. Kahn

**Affiliations:** Division of Endocrinology, Diabetes and Metabolism, Department of Medicine, Beth Israel Deaconess Medical Center and Harvard Medical School, Boston, Massachusetts, United States of America; University of Texas Health Science Center at San Antonio, United States of America

## Abstract

Hypoglycemia and neuroglucopenia stimulate AMP-activated protein kinase (AMPK) activity in the hypothalamus and this plays an important role in the counterregulatory responses, i.e. increased food intake and secretion of glucagon, corticosterone and catecholamines. Several upstream kinases that activate AMPK have been identified including Ca^2+^/Calmodulin-dependent protein kinase kinase (CaMKK), which is highly expressed in neurons. However, the involvement of CaMKK in neuroglucopenia-induced activation of AMPK in the hypothalamus has not been tested. To determine whether neuroglucopenia-induced AMPK activation is mediated by CaMKK, we tested whether STO-609 (STO), a CaMKK inhibitor, would block the effects of 2-deoxy-D-glucose (2DG)-induced neuroglucopenia both *ex vivo* on brain sections and *in vivo*. Preincubation of rat brain sections with STO blocked KCl-induced α1 and α2-AMPK activation but did not affect AMPK activation by 2DG in the medio-basal hypothalamus. To confirm these findings *in vivo*, STO was pre-administrated intracerebroventricularly (ICV) in rats 30 min before 2DG ICV injection (40 µmol) to induce neuroglucopenia. 2DG-induced neuroglucopenia lead to a significant increase in glycemia and food intake compared to saline-injected control rats. ICV pre-administration of STO (5, 20 or 50 nmol) did not affect 2DG-induced hyperglycemia and food intake. Importantly, activation of hypothalamic α1 and α2-AMPK by 2DG was not affected by ICV pre-administration of STO. In conclusion, activation of hypothalamic AMPK by 2DG-induced neuroglucopenia is not mediated by CaMKK.

## Introduction

The AMP-activated protein kinase (AMPK) is an evolutionarily conserved enzyme that senses cellular energy status and regulates fuel availability [1]. AMPK acts as an energy and glucose sensor in the hypothalamus to control food intake and metabolism in peripheral tissues. AMPK is a heterotrimeric protein consisting of catalytic α- and regulatory β- and γ- subunits. AMPK is activated allosterically by increases in the AMP/ATP ratio as well as by phosphorylation on Thr^172^ by upstream kinases [1] while ADP allosterically protects AMPK from dephosphorylation and inactivation [2], [3].

Presently, three upstream AMPK Kinases (AMPKK) have been identified, Liver Kinase B1 (LKB1), Ca^2+^/Calmodulin-dependent protein kinase kinase (CaMKK) and transforming growth factor-beta-activated kinase (TAK1) [4], [5], [6]. The CaMKK family is composed of two isoforms CaMKKα and CaMKKβ. Since the original findings that CaMKKβ phosphorylates and activates AMPK via an AMP-independent manner, which is triggered instead by a rise in intracellular Ca^2+^ concentration [5], [7], [8], many studies have demonstrated that AMPK is activated by CaMKK in peripheral tissues or cells in response to nutrients, drugs, or physiological stimulation [9], [10], [11], [12], [13]. CaMKKα and CaMKKβ are highly expressed throughout the brain including in several areas controlling food intake and neuroendocrine function (i.e. hypothalamus and hindbrain) [14], [15] suggesting that CaMKKs could play an important role in the regulation of AMPK activity and control of energy balance. In line with this, recent data demonstrated that CaMKKβ is involved in ghrelin-induced AMPK activation in the hypothalamus as well as in the orexigenic action of ghrelin [15].

Several studies have demonstrated that hypothalamic AMPK is regulated by blood glucose levels. Peripheral or central hyperglycemia inhibits AMPK in several hypothalamic nuclei, the arcuate nucleus (ARC), the ventromedial hypothalamus (VMH)/dorsomedial hypothalamus (DMH), the paraventricular nucleus (PVN) and the lateral hypothalamus (LH) [16], [17] whereas insulin-induced hypoglycaemia or 2-deoxy-D-glucose (2DG)-induced glucopenia activates AMPK [16], [18], [19]. Recent studies suggest that activation of hypothalamic AMPK may be required for the counterregulatory response to hypoglycemia [18], [19], [20]. Moreover, hypothalamic AMPK activation causes an increase in food intake, which may act to restore depleted energy stores [16], [18], [19]. However, it is still unclear which upstream kinases are involved in hypothalamic AMPK activation in response to metabolic challenges such as neuroglucopenia.

The primary goal of the present study was to assess whether CaMKKs are involved in the hypothalamic activation of AMPK by neuroglucopenia. In this study, we incubated brain slices *ex-vivo* in the presence of KCl (to induce neuronal depolarization) or 2DG with or without STO-609, an inhibitor of CaMKKs. Furthermore, we investigated the effect of STO-609 on the counterregulatory responses to neuroglucopenia induced *in vivo* by intracerebroventricular (ICV) administration of 2DG.

## Materials and Methods

### Animals

Male Sprague-Dawley rats (Charles River Laboratories, Wilmington, MA) were housed one per cage with a constant temperature (21–23°C) and a 14∶10 h light/dark cycle with access to food and water *ad libitum.* All study protocols were approved by the Institutional Animal Care and Use Committee (Beth Israel Deaconess Medical Center).

### Western Blot Analysis

Tissue lysates were prepared as described previously [19]. The total amount of CaMKKα and CaMKKβ protein in hypothalamic nuclei and different brain areas was determined using 10% SDS acrylamide gels and antibodies specific for CaMKKα or CaMKKβ (Santa Cruz Biotechnology, Santa Cruz, CA).

### Preparation and Incubation of Rat Brain Slices

Coronal hypothalamic slices were prepared from 7-week-old male Sprague-Dawley rats. Following decapitation, the brain was rapidly removed and immersed in high-Mg^2+^ but Ca^2+^-free, ice-cold artificial cerebrospinal fluid (aCSF) of the following composition (mM): NaCl, 114; KCl, 3; NaH_2_PO_4_, 1.25; MgSO_4_, 1; MgCl_2_, 10; Hepes-Na (pH 7.4), 10; NaHCO_3_, 26; D-glucose, 10; pH 7.4; bubbled with 95% O_2_-5% CO_2_. Three hypothalamic sections, each 400 µm thick, were cut from each rat using a Vibratome while being continuously immersed in ice-cold aCSF. Hypothalamic sections were maintained in an incubation chamber at room temperature for 30 min and then at 36°C for 2 hr with standard aCSF (2 mM CaCl_2_ instead of 10 mM MgCl_2_). After the pre-incubation period, sections were pre-treated for 30 min with either vehicle (100 µM NaOH) or STO-609 (25 µM, Tocris, Ellisville, MO). KCl (30 mM) was added in the experimental chambers for 5 min to induce neuronal depolarization. Glucopenia was induced by incubating the sections with 10 mM D-glucose or 10 mM 2DG for 15 min. After the incubation, the medio-basal region of the hypothalamus including the ARC and VMH/DMH was dissected as described [19] and snap frozen in liquid nitrogen. Samples were stored at −80°C until subsequent homogenization and AMPK activity assay. Each treatment was repeated with sections from at least five different rats.

### Lateral Ventricle Cannulation

Male Sprague-Dawley rats (Charles River), weighing 300–350 g, were stereotaxically implanted with a 26-gauge stainless steel cannula (Plastics One, Roanoke, VA) aimed at the right lateral ventricle as described previously [19].

### Intracerebroventricular (ICV) Injections

For this experiment, fed rats were assigned to three different groups. A control group was injected ICV with saline 30 min before an ICV saline injection (Saline-Saline). The Saline-2DG group was injected ICV with saline 30 min prior to an ICV 2DG injection. The STO-2DG group was injected ICV with STO-609 30 min before the ICV injection of 2DG. The ICV injections were performed as follow. The ICV injections of saline or STO-609 (5, 20 or 50 nmol), at a speed of 0.5 µl/min in 10 min using microdialysis pumps, were made using a 31-gauge injector (equal length of the cannula). 30 min after saline or STO-609 ICV injection, saline or 7 mg (40 µmol) of 2DG was injected ICV at a speed of 10 µl in 3 min using microdialysis pumps [19]. The injector was kept in place for an additional minute before it was removed and replaced by the dummy cannula. Plasma glucose was measured using the One-Touch Ultra glucometer from the tail vein vessels before (0 min) and 60 min after 2DG ICV injection. One hour after 2DG injection, a pre-weighed quantity of food was introduced in the cage and food intake was measured over 1 hour. For the AMPK activity experiments, the animals received saline or STO-609 (50 nmol) ICV injections as described above and were killed by decapitation 10 minutes after saline or 2DG ICV injection. Immediately after the decapitation, ARC and VMH/DMH were dissected as described [19] and frozen in liquid nitrogen.

### AMPK Activity Assay

AMPK activity was measured in hypothalamic regions by immunoprecipitation of α1-AMPK or α2-AMPK from hypothalamic regions (50 µg) with specific antibodies against the catalytic α1-subunit or α2-subunit (generous gift from Dr. D. Carling) bound to protein-G/Sepharose beads. Kinase activity was measured using synthetic “SAMS” peptide and [γ-^32^P] ATP as described previously [17].

### Data Analysis

All data are presented as means ± SEM. Significance is set at p<0.05. Analyses of α1-AMPK or α2-AMPK activities in hypothalamic samples were performed by two-way ANOVA. Comparisons of α1-AMPK or α2-AMPK activity in hypothalamic nuclei after 2DG injection *in vivo* were made by unpaired t-test. Analyses of glycemia and food intake were performed using two-way ANOVA.

## Results

### Expression of CaMKKα and CaMKKβ in Hypothalamic Nuclei

**Figure 1:**
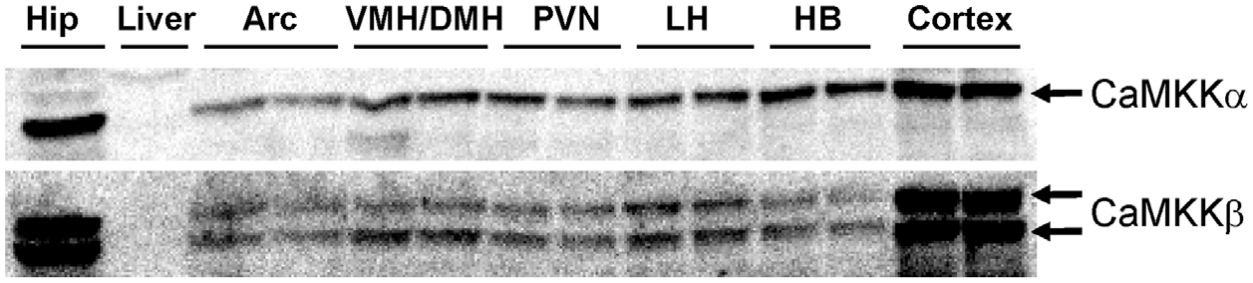
CaMKKα and CaMKKβ protein expression in hypothalamic nuclei and non-hypothalamic brain areas. Tissue lysates (40 µg protein) were subjected to Western blotting with anti-CaMKKα or anti-CaMKKβ antibodies (Santa Cruz) as described in “Materials and Methods”. Hip; hippocampus, Arc; arcuate nucleus, VMH/DMH; ventromedial hypothalamus/dorsomedial hypothalamus, PVN; paraventricular nucleus, LH; lateral hypothalamus, HB; hindbrain.

In agreement with previous studies performed in rats [14], [19], CaMKKα and CaMKKβ proteins were detected in all hypothalamic nuclei analyzed and in hindbrain. However, the protein levels in these regions were lower than the levels in cortex and hippocampus (Fig. 1). CaMKKα and CaMKKβ were undectable in liver protein extracts.

### Hypothalamic AMPK Activation by K(+)-Induced Depolarization is Blocked by STO-609 in Brain Sections Ex-vivo

**Figure 2:**
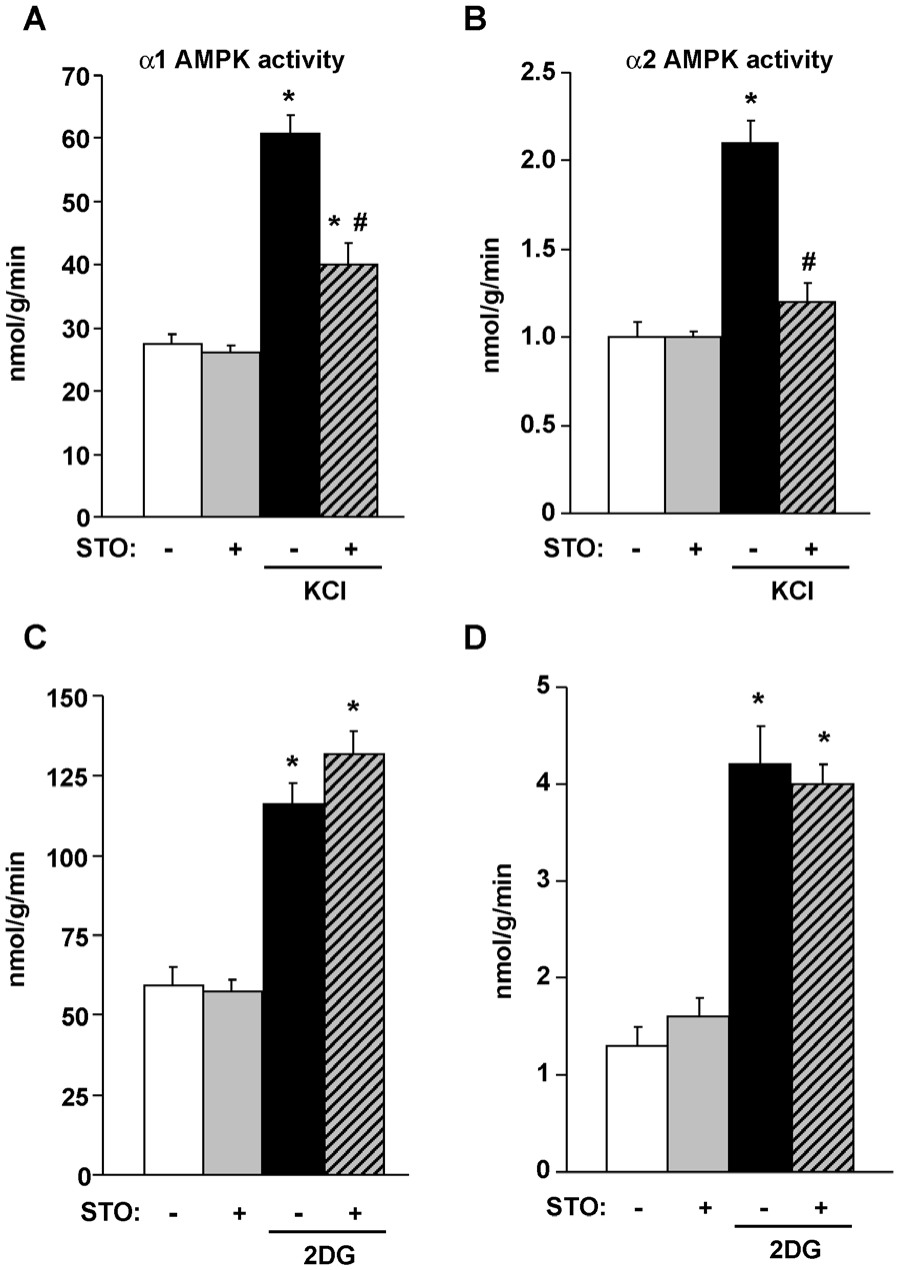
STO-609 blocks AMPK activation induced by KCl but not by 2DG in hypothalamus. Brain sections, pre-incubated with either vehicle (100 µM NaOH) or STO-609 (25 µM) for 30 min, were treated with or without KCl (30 mM) for 5 min (A and B). Brain sections, pre-incubated with either vehicle (100 µM NaOH) or STO-609 (25 µM), an inhibitor of CaMKKs, for 30 min were treated with D-glucose (10 mM) or 2DG (10 mM) for 15 min (C and D). α1 (A and C) or α2 AMPK (B and D) activities were measured in Arcuate-VMH/DMH samples. n = 5−6 rats per group. Data are means ± SEM. *p<0.05 vs. control, #*p*<0.05 vs. KCl alone.

Brain sections, pre-incubated with either vehicle (100 µM NaOH) or STO-609 (25 µM) for 30 min, were treated with or without KCl (30 mM) for 5 min to induce neuronal depolarization.STO-609 treatment did not affect basal α1-AMPK or α2-AMPK activities (Fig. 2A and B) in the medio-basal hypothalamus. K^+^-induced depolarization, which increases intracellular calcium and activates CaMKKs pathways in cells [5], [7], [8], resulted in a 2.2 fold-increase in α1-AMPK and a 2.1 fold-increase in α2-AMPK activity in the hypothalamus. STO-609 pre-treatment partially inhibited (66%) K^+^-induced α1 AMPK activation (Fig. 2A) and totally inhibited K^+^-induced α2 AMPK activation (Fig. 2B). These data are in agreement with a previous study [5].

#### Hypothalamic AMPK activation by 2DG is not inhibited by STO-609 in a brain section preparation

After pre-incubation with either vehicle or STO-609 (25 µM) for 30 min, brain sections were incubated in presence of 10 mM D-glucose or 10 mM 2DG for 15 min. 2DG treatment induced a 2 fold-increase in α1-AMPK activity and a 3.2 fold-increase in α2-AMPK in the hypothalamus (Fig. 2C and 2D). In contrast to K^+^-induced AMPK activation, 2DG-induced α1 and α2-AMPK activation were not inhibited by STO-609 pre-treatment (Fig. 2C and 2D).

#### Counterregulatory responses to 2DG-induced neuroglucopenia are not affected by STO-609

**Figure 3:**
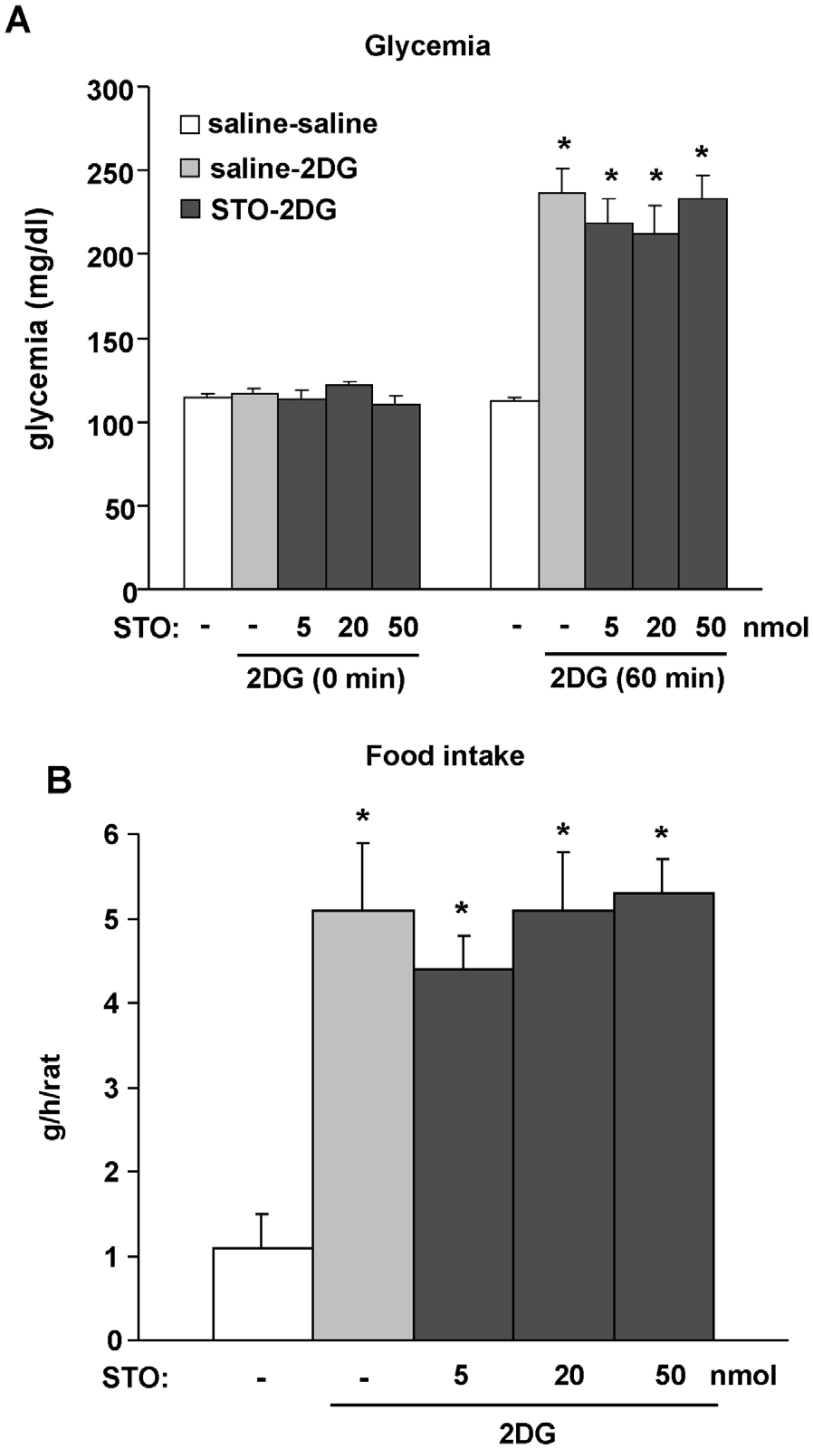
STO-609 does not affect counter-regulatory responses to neuroglucopenia *in vivo*. Saline or STO-609 (5, 20 or 50 nmol) was injected ICV in fed rats 30 min before ICV injection of saline or 2DG (40 µmol) as described in “Materials and Methods”. Glycemia (A) and 1-h food intake (B) were measured. n = 5−9 rats per group. Data are means ± SEM. *p<0.05 vs. saline-saline group.

ICV injection of 2DG was performed to induce glucopenia specifically in the brain [19]. The main advantage of using ICV 2DG compared with insulin-induced hypoglycemia is that the 2DG effects are acutely localized to the brain so that one can study signalling in the hypothalamus in the absence of other potentially confounding systemic effects that could modify the effects in the brain. We used a 2DG concentration known to induce glucopenia that is rapidly followed by stimulation of food intake and counterregulatory responses including increased plasma corticosterone and glucagon levels [19]. Rats were divided in to 3 groups, Saline-Saline group (control), Saline-2DG group (Saline-2DG) and STO-2DG group (2DG-STO) as described in “Materials and Methods”. To determine the optimal dose of STO609 ICV, we injected rats with different doses of STO (5, 20 or 50 nmol) in the lateral ventricle 30 min before 2DG ICV. Basal glycemia (0 min) was not different among the three animal groups (Fig 3A). ICV 2DG elicited a significant increase in glycemia 60 min after the injection in the Saline-2DG group compared to the Saline-Saline group (Fig 3A). Consistent with the glycemia data, 2DG injection induced a 5-fold increase in food intake over 1 h compared with the Saline-Saline group (Fig 3B). In the STO-2DG group, ICV STO-609 pre-treatment (5, 20 or 50 nmol) did not affect the 2DG-induced increase in glycemia 60 min after 2DG injection (Fig 3A) nor the 2DG-induced increase in 1-h food intake compared to the Saline-2DG group (Fig 3B).

### AMPK Activation by Neuroglucopenia is not Affected by STO-609

**Figure 4:**
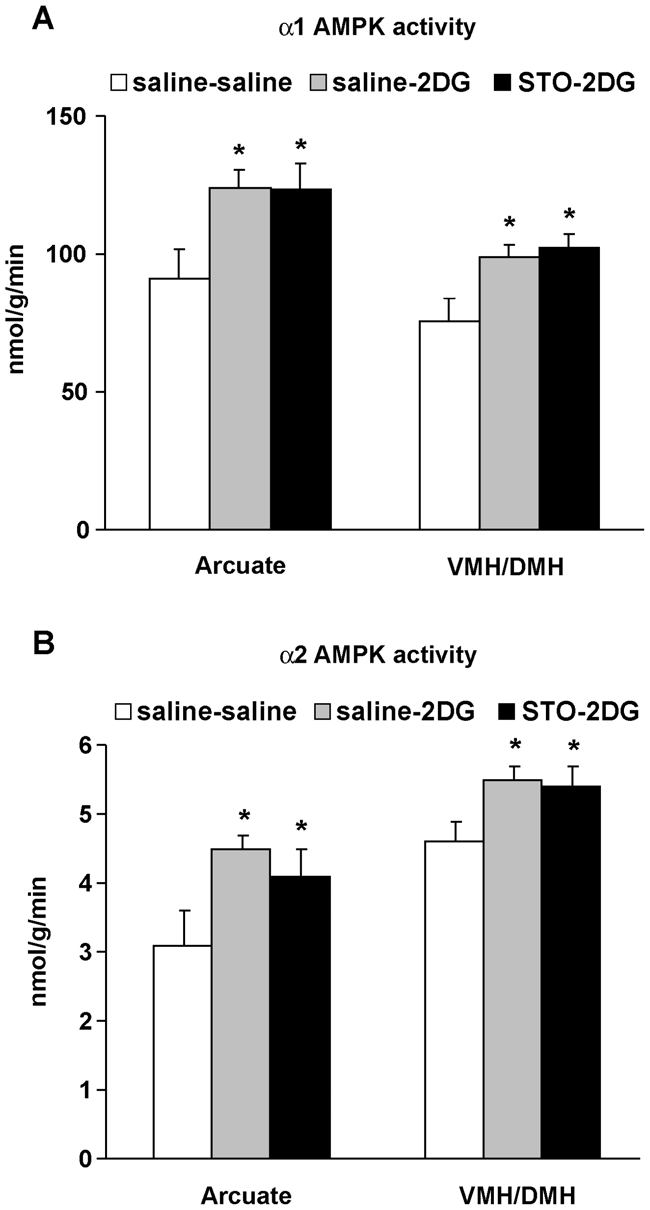
STO-609 does not block 2DG-induced AMPK activation in ARC and VMH/DMH. Saline or STO-609 (50 nmol) was injected ICV in fed rats 30 min prior to saline or 2DG (40µmol) ICV injection. Hypothalamic nuclei were dissected 10 min after saline or 2DG injection. α1 (A) and α2 (B) AMPK activities were measured in microdissected Arcuate and VMH/DMH nuclei. n = 9 rats per group. Data are means ± SEM. *p<0.05 vs. saline-saline group.

Both α1 and α2-AMPK activities were measured in ARC and VMH/DMH hypothalamic areas 10 min after 2DG injection in rats pre-treated with saline or STO-609 (50 nmol). 2DG ICV injection elicited a 40% increase in both α1 and α2-AMPK activities in the ARC and a 25% increase of α1 and α2-AMPK activities in the VMH/DMH 10 min after the injection compared to Saline-Saline group (Fig 4A and B) as described previously [19]. However, STO-609 pre-treatment did not affect the 2DG-induced α1 and α2-AMPK activation in the ARC or VMH/DMH (Fig. 4A and B).

## Discussion

AMPK is activated allosterically by increases in the AMP/ATP ratio as well as by phosphorylation on Thr^172^ by upstream kinases including LKB1 and CaMKKs [1] while ADP prevents its dephosphorylation and inactivation [2], [3]. Several studies have recently suggested a physiologic role for CaMKKα and CaMKKβ in AMPK activation in mamalian cells [5], [7], [8], [15]. CaMKKβ null mice ate less and accumulated less body weight and fat stores supporting the role of central CaMKKβ in the control of energy balance [15]. In the central nervous system, CaMKKs are involved in different processes including brain development, neuron survival and long-term memory. However, the hypothesis that CaMKKs could be involved in AMPK activation in the hypothalamus in response to glucopenia *in vivo* has not been tested. In this study, we demonstrate that hypothalamic AMPK activation by neuronal depolarization is CaMKKs dependent whereas glucopenia-induced AMPK activation in the same hypothalamic regions does not involve the CaMKK pathway.

Consistent with a previous report [14], we showed that both CaMKKα and CaMKKβ are expressed in several hypothalamic nuclei as well as the hindbrain. However, CaMKKα and CaMKKβ expression levels in those areas are lower compared to cortical or hippocampal expression levels. Using a brain section model *ex-vivo*, we demonstrated that neuronal K^+^-induced depolarization triggers hypothalamic AMPK activation and that pre-treatment with the CaMKK inhibitor STO-609 prevents depolarization-induced α1 and α2-AMPK activation in the medio-basal hypothalamus (ARC and VMH/DMH). These data confirm a previous report showing that CaMKK inhibition by STO-609 on brain slices inhibits depolarization-induced AMPK activation [5]. However, in that study total AMPK activity (α1 and α2-AMPK) was measured in a whole brain slice protein extract. Thus, we demonstrated here that CaMKK pathway inhibition by STO-609 blocks the K^+^-induced activation of both α1 and α2-AMPK in hypothalamic nuclei (ARC and VMH/DMH).

To determine whether the CaMKK pathway is also required for hypothalamic AMPK activation during glucose deprivation, brain slices were incubated in the presence of the glucoprivic agent 2DG to induce glucopenia. We previously reported that 2DG-induced glucopenia *in vivo* rapidly activates AMPK in several hypothalamic nuclei including the ARC and VMH/DMH [19]. In brain sections *ex-vivo*, 2DG treatment triggers activation of both α1 and α2-AMPK in the hypothalamus. However, in contrast to K^+^-induced activation of AMPK, STO-609 did not inhibit activation of hypothalamic α1 or α2-AMPK in response to 2DG. These data strongly suggest that activation of hypothalamic AMPK by glucoprivation is not CaMKK dependent. Despite the fact that both stimuli activate AMPK, our results suggest that the signalling pathways leading to AMPK activation are different. This difference could be explained in part by the nature of the intracellular signals modulated by 2DG and K^+^-induced depolarization. Indeed, it is well established that both K^+^-induced depolarization and 2DG-induced glucopenia result in an increase in intracellular calcium in neuronal cells [21]. However, K^+^-induced depolarization does not affect the ATP/AMP ratio [5] whereas 2DG increases the ATP/AMP ratio in neuronal cells [22]. From these observations and our data, we hypothetize that the increase in the ATP/AMP ratio induced by 2DG activates AMPK independently of the CaMKK pathway and that another AMPK upstream kinase might be involved in 2DG activation of AMPK such as LKB1 [23].

To support our *ex-vivo* findings on brain sections, we tested the effect of the CaMKK inhibitor *in vivo* on the counterregulatory responses to neuroglucopenia induced by 2DG. We previously reported that 2DG ICV injection in rat brain rapidly activates the counterregulatory responses (increases in food intake and counterregulatory hormones) and triggers both α1 and α2-AMPK activation in several hypothalamic nuclei (i.e. ARC and VMH/DMH) [19]. We and others also showed that hypothalamic AMPK activation by neuroglucopenia or insulin-induced hypoglycemia is required for the counterregulatory responses [18], [19], [20]. As demonstrated, ICV 2DG induces counterregulatory responses to neuroglucopenia resulting in marked increases in blood glucose levels and food intake. However, these responses were not affected by pre-treatment with STO-609. Consistent with these findings, 2DG-induced activation of α1 and α2-AMPK in ARC and VMH/DMH after 2DG injection was not affected by prior treatment with STO-609. These data suggest that the CaMKK pathway is not involved in the activation of hypothalamic AMPK and the couterregulatory response to glucopenia. The STO-609 concentrations used *in vivo* were extrapolated from the concentrations used *ex-vivo* on brain slices (based on a rat CSF volume of 150 µl). Because STO-609 is a cell-permeable inhibitor, we used a 10-fold range of STO-609 doses. We can not rule out the possibility that STO-609 only partially inhibited CaMKKs when injected ICV. However, our *ex-vivo* data combined with the *in vivo* findings strongly suggest that AMPK activation in the hypothalamus by glucopenia is not CaMKKs dependent. Thus, we conclude that the signaling pathways involved in AMPK activation in the hypothalamus are dictated by the nature of the stimulus and the intracellular signals involved (intracellular calcium vs. ATP/AMP and ATP/ADP ratios). These findings are supported by the work of Anderson et al. showing that the orexigenic action of ghrelin, which elicits increases in neuronal intracellular calcium, is absent in CaMKKβ null mice whereas the orexigenic action of 2DG was not affected [15]. However, the counterregulatory response *per se* (hyperglycemia) to 2DG was not measured in that study. Therefore, our results also demonstrate that 2DG-induced hyperglycemia is CaMKK independent.

In summary, the CaMKKs inhibitor STO-609 did not block hypothalamic AMPK activation induced by glucopenia in rats or in brain slices *ex vivo*, whereas the inhibitor blunted the activation of hypothalamic AMPK by K^+^-induced depolarization of brain slices *ex vivo*. ICV administration of STO-609 *in vivo* did not affect the counterregulatory responses to neuroglucopenia and AMPK activation induced by glucopenia. We conclude that AMPK is activated in hypothalamic nuclei by neuroglucopenia via a CaMKK-independent pathway.
